# External exposure assessment in the Fukushima accident area for governmental policy planning in Japan; Part 2. Matters to be attended for assessments of external exposure

**DOI:** 10.1093/jrr/rrac088

**Published:** 2023-01-04

**Authors:** Kazuya Yoshimura, Yukihisa Sanada, Rina Sato, Mariko Nakayama, Masaharu Tsubokura

**Affiliations:** Sector of Fukushima Research and Development, Japan Atomic Energy Agency, Minamisoma-shi Fukushima 975-0036, Japan; Sector of Fukushima Research and Development, Japan Atomic Energy Agency, Minamisoma-shi Fukushima 975-0036, Japan; Hitachi Solutions East Japan, Ltd., Sendai-shi Miyagi 980-0021, Japan; PESCO Co., Ltd., Minato-ku Tookyo 105-0021, Japan; Department of Radiation Health Management, Fukushima Medical University, Fukushima-shi Fukushima 960-1295, Japan

**Keywords:** governmental policy, Fukushima Daiichi Nuclear Power Plant (FDNPP) accident, external exposure assessment, uncertainty, points to be considered

## Abstract

After the Fukushima Daiichi Nuclear Power Plant (FDNPP) accident, individual exposure doses to residents have been assessed by many municipalities, governments and research institutes. Various methods including measurements with personal dosimeters and simulations have been used for this evaluation depending on purposes, but the information of assessments and methods has not been systematically organized. A comprehensive review of the knowledge and experiences of individual exposure doses assessments accumulated so far and understanding the characteristics of the assessment methods will be very useful for radiation protection and risk communication, following to governmental policy planning. We reviewed the efforts made by the Japanese government and research institutes to assess radiation doses to residents after the FDNPS accident in Part 1. On the other hand, each method of assessing individual exposure doses includes uncertainties and points to be considered for the appropriate assessment. These knowledge and experiences are important for the assessment implementation and applying the assessment results to the governmental policy planning, and are summarized in Part 2 of this article.

## INTRODUCTION

Due to the accident at the Fukushima Daiichi Nuclear Power Plant (FDNPP) of the Tokyo Electric Power Company (TEPCO) Holdings, Incorporated in March 2011, ambient dose rates increased over a wide area centered on Fukushima Prefecture, and evacuation order zones were established accordingly. Thereafter, the evacuation order zones have been reorganized according to decrease in air dose rate with time and progress in decontamination.

Upon the reorganization of the evacuation order zones and the lifting of the evacuation order, the Nuclear Regulation Authority (NRA) compiled the *Basic Approach to Safety and Security Measures for Return to Home (For Specific Implementation of Protective Measures Based on Dose Levels)* (November 2013) to reduce radiation doses of residents and address their concerns about the health effects of radiation. In this policy, the importance of assessment, risk management and risk communication to residents based on individual exposure doses were mentioned. As described in Part 1, many local and central governments have measured the individual exposure doses of residents using personal dosimeters such as Fukushima Restoration Acceleration Grant Project [1] and the Fukushima Health Management Survey [2] since the FDNPS accident. In addition, individual exposure doses also have been evaluated based on simulations such as Monte Carlo simulations [3] and probabilistic assessment [4] in addition to estimation conducted by international organizations [5, 6], because dosimeters are difficult to measure individual exposure doses continuously throughout the year and to evaluate predictively. The individual exposure doses obtained by these evaluations have been utilized by governments as effective information, not only for radiation protection measures but also for reducing the anxiety of residents about health effects due to radiation. The knowledge and experience, regarding individual exposure dose assessments, accumulated after the FDNPS accident are important for the application of the assessments for the future lifting of evacuation order zone.

Studies and projects of individual exposure dose assessments carried out after the FDNSP and Chernobyl Nuclear Power Station (CNPS) accidents have been reviewed at different viewpoints. Lopez *et al.* surveyed individual monitoring data and dose assessment for foreigners exposed in Japan and summarized issues of dosimetry and dose assessments [7]. Methodological findings and approaches of retrospective dosimetry after the CNPS accident were reviewed, and applicability to the case in Fukushima was proposed [8]. Studies and projects of individual exposure dose estimations using personal dosimeters and simulations after the FDNSP accident were also reviewed, and some uncertainty was described [9]. However, there is no comprehensive and systematic review of the various methods used in projects and studies by municipalities, governments and research institutions.

We aimed to review the methodological and operational knowledge and experience, regarding individual exposure dose assessments conducted by diverse organizations, accumulated after the FDNPS accident. Part 1 reviewed the methods of individual exposure dose assessments to residents made by the Japanese government and research institutes after the FDNPS accident. The methods, including both actual measurements and simulations, were categorized by time axis (i.e. retrospective or predictive) and targets (i.e. individual or population), and their characteristics and applicability to the purposes of governmental policy were summarized in Part 1. On the other hand, there are uncertainties and points to be considered for each method. It is essential to understand these uncertainties and points to conduct individual exposure dose assessments for risk communication, radiation protection and governmental policy planning in the future, and are summarized in this article, Review Part 2.

## UNCERTAINTIES FOR ASSESSMENT USING PERSONAL DOSIMETERS

The personal dose equivalent described in this chapter refers to the results of measurements using personal dosimeters. As mentioned in Part 1, the effective dose is required as a protection quantity representing the individual exposure dose for radiation protection. In the case of rotational irradiation geometry, which is analogous to a situation in which radionuclides are deposited in the environment, the personal dose equivalent is close to the effective dose [10]. Therefore, the personal dose equivalent is often regarded as an approximation of the individual exposure dose after the FDNPS accident. However, it should be noted that the personal dose equivalent and the effective dose are not the same.

### Characteristics of personal dosimeters

Personal dosimeters can be classified as passive or active. Passive personal dosimeters measure the accumulated dose over a certain time period. They are durable, reliable and can be used for long periods over a month since they do not require electricity, however, the personal dose equivalent cannot be confirmed in real-time. Passive personal dosimeters include fluorescent glass dosimeters and optically stimulated luminescence dosimeters. Active personal dosimeters are capable of obtaining personal dose equivalents at regular time intervals. Trends over time can be determined and many can provide real-time personal dose equivalents. However, most active personal dosimeters are susceptible to physical shocks and electromagnetic noise. Furthermore, they are unsuitable for long-term use because they require electricity. It is necessary to understand the characteristics of each device and select the type of personal dosimeter according to its purpose.

Personal dosimeters are calibrated to the personal dose equivalent, *H*p(10), but responses to irradiation differ among the personal dosimeters. The National Institute of Radiological Sciences and the Japan Atomic Energy Agency compared five types of electronic personal dosimeters [11]. The personal dosimeters were attached to three different phantoms, each of which was irradiated in rotation using a ^137^Cs gamma-ray standard irradiation field. They reported differences in personal dose equivalents of 14%–25% among the personal dosimeters. This difference was attributed to the different sensitivities, depending on energy and the direction of radiation for individual dosimeters. Therefore, it is necessary to consider the uncertainties of personal dosimeters when selecting instruments and analyzing the results.

### Dosimeter mounting

To measure the personal dose equivalent properly, personal dosimeters must be worn correctly at the position specified by the manufacturer of the dosimeter. However, wearing a personal dosimeter continuously throughout the day is difficult, particularly during bathing and sleeping time. The Ministry of the Environment’s ‘Guidelines for Measurement of Personal Exposure Doses of Residents after the Accident at Tokyo Electric Power Company’s (TEPCO) Fukushima Daiichi NPP and Handling of Results, etc.’ states that personal dosimeters should be kept as close as possible to the person being measured during times when they cannot be worn [12]. Nomura *et al*. surveyed personal radiation doses among elementary and junior high school students in Minamisoma and reported that the data from students who wore personal dosimeters throughout the day was only 7% [13]. They reported no statistically significant difference between data obtained with correct fittings and with faulty fittings. However, if the personal dosimeter is not worn on the body, the personal dose equivalent can be higher than the actual value due to the lack of shielding effect by the body. Therefore, considering the uncertainty of individual doses, it is recommended to pay attention to the mounting situation and to record the wearing status for use in validation of the data.

## POINTS TO BE CONSIDERED FOR ASSESSMENT USING PERSONAL DOSIMETERS

### Radiation from natural radionuclides

Measured personal dose equivalents include the radiation from natural radionuclides (hereafter referred to as background dose). To determine the personal dose equivalents due to the nuclides derived by the accident, the background dose must be subtracted from the personal dose equivalent. Since the background dose varies among regions, it is ideal to apply the background dose from the measurement area for subtraction. However, it is difficult to identify the background dose at each location in the measurement of personal dosimeters. The common method for identifying the background dose at each location is to use control badges, but this approach assumes that personal dosimeters were used to assess the occupational exposure before the accident, and is not optimized for assessing personal dose equivalent of residents over a large area. For this reason, background values obtained by clarifying assumptions and conditions for different types of personal dosimeters are used. As an example, passive personal dosimeters such as Glass Badge (Chiyoda Technol Corporation) and Luminescence Badge (Nagase Landauer, Ltd.) use a value obtained by control badge stored at each business site in a location unaffected by radiation as a background. In the absence of control badges or after an accident where it is difficult to distinguish between background and additional exposure doses, the criteria of personal dosimeter manufacturers are often used.

### Noise in obtained data

Noise effects also require attention in evaluating the measurement results of personal dosimeters. Because electronic personal dosimeters are susceptible to physical shocks and electromagnetic noise, extremely large peaks may be found in the measured personal dose equivalents, but it is difficult to identify the cause of such peaks. The Ministry of the Environment’s ‘Guidelines for Measurement of Personal Exposure Doses of Residents after the Accident at TEPCO’s Fukushima Daiichi NPP and Handling of the Results’ states that peaks should not be excluded as noise when the cause cannot be identified for a safe side evaluation [12]. In many cases, the peaks are of short duration and contribute little to the cumulative doses; therefore, there is no significant impact if the cumulative doses are tabulated.

### Behavioral records as supplementary information

The behavior of residents is a factor to affect the personal dose equivalents. Takahara *et al*. reported that the time spent outdoors varies with occupation and season, and personal dose equivalents fluctuate due to the variation of the time spent outdoors [4]. The UNSCEAR simulated individual exposure doses arranging a parameter named occupancy factors, which is the fraction of time spent in the various location types, depending on age and occupation (outdoor and indoor workers) [5]. Therefore, it is useful to record the behavior (i.e. time spent outdoors) to evaluate the representativeness of measured personal dose equivalent. In addition, evaluation of behavioral records (i.e. the location and time of stay) is effective in determining the time and location critically affect to the personal dose equivalent, meaning the records is useful for measures to reduce personal dose equivalents.

## UNCERTAINTIES FOR SIMULATIONS

The estimation of individual exposure doses in this chapter refers to the simulations based on parameters such as ambient dose rates, etc. As mentioned in Part 1, it is desirable to obtain effective doses, which are protective quantities, as individual exposure doses when radiation protection is taken into account. The effective dose can be estimated from monitoring quantities such as the ambient dose rates. An example of an expression for effective dose (*E*) is shown in equation (1):


1 \begin{equation*} \boldsymbol{E}=\sum \boldsymbol{c}\cdot \dot{\boldsymbol{D}}\left(\mathbf{p}\right)\cdot \boldsymbol{t}\left(\mathbf{p}\right) \end{equation*}

where *c* is the conversion factor from the measurable dose to the effective dose, *D* ˙(*p*) is the measurable dose rate at location (*p*), and *t*(*p*) is time spent at location (*p*). The *D* ˙(*p*) includes the ambient dose rates as well as the personal dose equivalents, as mentioned in Part 1, but the *D* ˙(*p*) in this chapter on simulations refers to ambient dose rates.

An estimation using ambient dose rates is the calculation method adopted by the Japanese government to estimate additional annual exposure doses in the early period after the FDNPS accident [14]. This calculation method assumes that a person stays outdoors for 8 h and indoors for 16 h at the same location, and that the indoor ambient dose rate is 0.4 times the outdoor ambient dose rate. This calculation provides the integrated values of ambient dose rates in the targeted area (i.e. ambient dose equivalent). This calculation can provide higher values than the actual individual exposure dose because it does not take the conversion factor (*c*) into account and the time spent outdoors is longer than the typical situation [5]. Therefore, this calculation is suitable for conservative and rapid estimation of individual exposure doses just after the accident. However, this method is difficult to apply to the estimation of realistic individual exposure doses.

As shown in equation 1, accounting for behavioral patterns, such as place and time of stay, conversion to the effective dose allows individual exposure doses to be estimated with a certain accuracy without long-term measurements using personal dosimeters [15, 16]. Additionally, this calculation makes it possible to predictive assessment of individual exposure dose for residents who return to the ‘specific reconstruction reproduction base area (SRR Zone)’. Table 1 summarizes the main parameters used for estimating the individual exposure doses, the available tools and the uncertainties associated with the parameters. The details of each parameter are described below.

**Table 1:** Uncertainties in personal dose estimation parameters and available tools

	Parameter	Available tools	Uncertainty
*t (p)*	Life behavior pattern	• Handwriting • GPS	• Inaccurately listed • Positioning accuracy
	Statistics of staying time	• General statistical surveys	• Population characteristics
*D (p)*	Ambient dose rate (outdoor)	• Actual measurement • Literature value	• Characteristic of measurement technique
	Ambient dose rate (indoor)	• Actual measurement • Reduction factor of buildings	• Uncertainty of reduction factor of buildings
	Background	• Literature value	• Position resolution
*c*	Conversion Factors for Personal Dose	• Literature value	• Applicability condition (physical constitution, posture, irradiation condition)

### Life behavior pattern

To evaluate individual exposure doses more accurately, it is necessary to establish detailed behavioral patterns (location and time of stay) of the subjects. However, in the case of evaluating individual exposure doses for a population, the methods using statistic (mean value, standard deviation, etc.) for determining the times spent outdoors and indoors is applicable such as probabilistic assessment [4].

The individual behavioral patterns can be collected by both questionnaires and GPS information. Questionnaires are widely used and have the advantage that various information can be obtained as needed. Questionnaires were also used in the basic survey of the Fukushima Prefectural Health Survey conducted by Fukushima Prefecture after the accident [17]. Since questionnaires can be used for both retrospective and predictive assessments, they are useful when making assumptions about the behavioral patterns of people after their return. However, questionnaires are time-consuming to fill out and have the uncertainty of being prone to entry errors.

GPS information refers to the location and duration of stay and is recorded using tools such as GPS loggers and smartphones [16]. Since this method records actual behavior, it can only be used for retrospective evaluation and is difficult to apply to the predictive evaluation. The uncertainty is that location information may not be obtained correctly due to obstructions, weather and other factors. In addition, GPS loggers and smartphones are difficult to collect other information such as identification of indoor and outdoor locations. The Cabinet Office and the NRA have developed dedicated applications that can automatically distinguish between indoor and outdoor locations and can record other information [18].

Statistics on behavioral patterns (especially duration of daily activities) are reported through ongoing, large-scale statistical surveys (the Basic Survey of Social Life of the Statistics Bureau of the Ministry of Internal Affairs and Communications and the National Living Time Survey of the NHK Broadcasting Culture Research Institute). Both surveys are conducted every five years to provide average times for individual activities such as sleeping, eating, working and housework. These data do not distinguish between indoor and outdoor activities, but by assuming each classified activity is either indoor or outdoor, the time spent indoors and outdoors can be calculated. Additionally, there are surveys of individual activities time for exposure assessment, such as Takahara *et al*. [4], NRA [18] and Hirose *et al*. [19]. The results of these surveys show that the time spent indoors varies depending on occupation, season and age. Therefore, these factors can be uncertainties when using statistics on behavioral patterns for the simulation of individual exposure dose for group.

### Ambient dose rate

The assessment of individual exposure doses by simulation requires indoor ambient dose rates as well as outdoor ambient dose rates. The uncertainties of the outdoor and indoor ambient dose rates were described below.

#### Outdoor ambient dose rates

Although the outdoor ambient dose rates used in the simulations can be obtained either from direct surveys or from publicly available databases, this section describes the use of databases. Since the accident, ambient dose rates have been measured by various methods. In the monitoring projects implemented by the NRA [20, 21], ambient dose rates have been continuously measured using fixed-point measurements, walking surveys, car-borne surveys, unmanned helicopter monitoring, and airborne monitoring and these data are summarized in a database [22]. These are used differently according to the purpose of monitoring and the characteristics of the measurement methods [23]. While walking surveys have high data accuracy and spatial resolution, it is difficult to measure wide areas. Car-borne surveys have lower accuracy and spatial resolution than walking surveys but can acquire data over a wide area on the ground. Airborne monitoring has lower accuracy and spatial resolution than other surveys but can survey large areas, including the area without roads. Thus, the uncertainty of air dose rate depends on spatial resolution and area covered by surveys.

In an NRA project, wide area ambient dose rate maps (hereafter referred to as ‘integrated maps’) with high accuracy and spatial resolution have been created utilizing the characteristics of each of these monitoring methods [20, 24]. The integrated map is considered suitable for use when estimating individual exposure doses because it was created to obtain values equivalent to walking surveys, which reflect ambient dose rates in living areas using Bayesian statistics.

#### Indoor ambient dose rates

Indoor ambient dose rates are mainly determined by indoor measurements or by estimations from outdoor ambient dose rates using a reduction factor (ratio of indoor ambient dose rate to outdoor ambient dose rate) [5, 25]. The average time spent indoors in a day is about 20 h [4, 19], which is significantly longer than the average time spent outdoors; hence, indoor ambient dose rates have a greater impact on the daily individual exposure dose. Therefore, when estimating indoor individual exposure doses with high accuracy, it is recommended that actual indoor ambient dose rate is used for simulations. But the ambient dose rate varies depending on location in a building, and the variation can be an uncertain [26].

The estimation of indoor ambient dose rates using reduction factors can be easily evaluated for many buildings in areas where the outdoor ambient dose rates are monitored. However, the reduction factors include a certain degree of uncertainty, since they vary depending on the shielding effect of the building, building size and the surrounding contamination conditions. The International Atomic Energy Agency and the other international organization show typical reduction factor values and ranges depending on the structure and layout of the building [5, 6, 27]. Since many buildings in Fukushima Prefecture (about 76% in 2018 statistics) are wooden [28], 0.4 is often used as a representative reduction factor in post-accident exposure assessments. However, it should be noted that 0.4 is a representative value, and the representative range is indicated as 0.2 to 0.5. Furthermore, a study conducted on wooden houses in Fukushima after the accident reported that the reduction factor changed before and after decontamination [25].

### Conversion factors

This section describes the conversion coefficients used for converting the ambient dose equivalent to the effective dose, as well as the conversion coefficients for converting the ambient dose equivalent to the personal dose equivalent. The energy dependency of the effective dose and personal dose equivalent varies depending on the positional relationship between the radiation source and the human body, and on the shielding effect of the human body. Therefore, differences in the conditions (irradiation, physique (age), posture, etc.) used for setting the conversion coefficients and the actual situation may cause uncertainty in the evaluation of individual exposure doses.

Saito *et al*. [29] determined a conversion factor (from ambient dose equivalent to effective dose) for adults of about 0.6 in a field where radio cesium was deposited. The International Commission on Radiological Protection [30] determined relationships between the effective dose and ambient dose equivalent for different ages, irradiation conditions and nuclides. These effective dose simulations are performed assuming a person is in a standing position. Assuming a plane source of radiation, the effective dose is at most 20–30% larger for a standing position than lying face-down or -up positions. Therefore, the conversion factor assuming a standing position can be used to make a conservative evaluation [29].

The conversion factor (from ambient to personal dose equivalent) for adults was determined through simulations to be 0.66 in a field where radio cesium was deposited [31]. The National Institute of Radiological Sciences and the Japan Atomic Energy Agency [11] reported that the ratio of the ambient dose equivalent to personal dose equivalent was about 0.6–0.7 based on a study in which personal dosimeters were attached to phantoms assumed to be adults. In this study, the personal dose equivalent measured in the environment by an adult male of standard build was about 0.7 times higher than the ambient dose equivalent. In addition, conversion coefficients (from ambient to personal dose equivalent) are presented for each age group, based on the results of experiments and simulations using phantoms and personal dosimeters for each age group [32].

## POINTS TO BE CONSIDERED FOR SIMULATIONS

### Background ambient dose rate

The background ambient dose rate due to natural radionuclides should be subtracted when determining the additional individual exposure dose derived from anthropogenic radionuclides deposited in the environment. In the government’s estimation of the individual exposure dose after the FDNPS accident, 0.4 μSv h^−1^ was used as the background ambient dose rate [14]. The background ambient dose rates are variable by regions because they depend on soil, rock composition and altitude. Background ambient dose rates for different regions have been evaluated by Andoh *et al*. [33] and Sanada *et al*. [34] using analysis of gamma-ray spectra obtained by car-borne and air-borne monitoring surveys, respectively, and they provided average values for each municipality or as 250 m mesh map data.

### Character of target group

As mentioned above, since the conversion coefficients and the time spent indoors and outdoors depend on age and occupation, it is necessary to understand the character of target groups to evaluate their individual exposure dose levels. For example, population percentages by region, age, sex, occupation, etc. can be obtained from the results of the census and other sources.

By clarifying the characteristics of the target group, a probabilistic evaluation of individual exposure doses is possible [3, 4]. For example, Takahara *et al.* [4] estimated the distribution of individual exposure doses for a population by assigning a distribution to the parameters, such as time spent indoors and outdoors in consideration of occupation and age.

### Representativeness

Even within the same area, individual exposure doses and each parameter used in the simulation have variations and distributions depending on the characteristics (e.g. target type of occupations, ages and areas) of the population as described above. Because it is difficult to evaluate the individual exposure doses of all residents living in the target area, the distribution of the individual exposure doses is estimated based on a sample data collected from the target population. Additionally, the parameters in the simulation is also estimated from a sample extracted from the target population. To select the sample for the estimation of individual exposure doses distribution of population or apply the parameters in the simulation, it is important to ensure the sample and parameters representativeness.

The sample can be extracted by either purposive sampling or random sampling. The random sampling method is used to obtain highly accurate estimates. Random sampling methods include simple random sampling, cluster sampling, multistage sampling and stratified sampling. The number of samples required for a sample survey can be determined by the allowable error, confidence level and population size. Here the allowable error is a metric that represents the deviation from the population, and the confidence level is a metric that represents the probability that the extracted sample results are within the allowable error. When the allowable error and confidence level are constant, the required number of samples does not change much when the population number exceeds a certain value. For example, if the allowable error is ±5%, the confidence level is 95% and the population value is 10 000, the required number of samples is 370. If the population value increases 10-fold, the required number of samples is 383, showing minor increase.

It is difficult to confirm the preliminary representativeness of the number and characteristics of the target population, in cases such as when estimating the exposure doses of returning residents after the evacuation order has been lifted. In this cases, it is useful to ensure the differences in the distribution between the target population and sample after the survey. For example, Nomura *et al*. confirmed that there is a correlation between individual exposure doses and ambient dose rates in residential areas, and ensured the representativeness of the sample using the distribution of ambient dose rates in the residential areas as an indicator [35].

## CONCLUSION

This review summarized knowledge and experiences, obtained after the FDNPS accident, concerning on the following uncertainties and points to be considered for the assessment of individual exposure doses.

- Since the characteristics of the equipment, measurement conditions and evaluation conditions are sources of uncertainty, measurements using personal dosimeters should be conducted with consideration given to their validity.
- Since the parameters used for the simulation of individual exposure doses have a certain degree of uncertainty, it is necessary to set appropriate parameters based on past findings and actual measurements, as well as to evaluate them in consideration of the uncertainty.
- Additional information, such as characteristics of data, behavioral records, characteristics of target group and representativeness, should be considered to evaluate individual exposure dose adequately and beneficially.
